# Use of Ceftazidime-Avibactam in the Treatment of Clinical Syndromes With Limited Treatment Options: A Retrospective Study

**DOI:** 10.7759/cureus.33623

**Published:** 2023-01-10

**Authors:** Sneha Radha, Anup R Warrier, Arun Wilson, Shilpa Prakash

**Affiliations:** 1 Infectious Diseases, Aster Medcity, Kochi, IND; 2 Clinical Pharmacy, Aster Medcity, Kochi, IND

**Keywords:** antimicrobial resistance, limited treatment option syndromes, multidrug resistant organism infection, carbapenem resistant organism infection, zavicefta, ceftazidime avibactam

## Abstract

Background

With rising trends of multi-drug organism infections and the limited availability of new antimicrobials, management of such cases has become a hassle for the clinician. Ceftazidime-Avibactam (CEF-AVI) is evolving as an effective alternative to polymyxins in the management of Carbapenem-Resistant Organisms (CRO) infections. The Food and Drug Administration (FDA) has approved CEF-AVI in a restricted group of clinical syndromes where the drug could have potential use.

Objective

The goal of this study was to evaluate the clinical outcome in terms of 14-day all-cause mortality and clinical cure at seven days in patients on CEF-AVI.

Methodology

A retrospective study was conducted on patients who received CEF-AVI in a period of one year in our hospital. Patients were included in the study if they have received CEF-AVI for more than one day of therapy (DOT) and samples from relevant sites have been sent for culture and sensitivity. Variables and outcomes were collected from the hospital information system and medical records.

Results

A total of 78 patients were included, 52 (66.7%) were started empirically on CEF-AVI while 26 (33.3%) were on targeted therapy. Out of the 78 patients, 43 patients had positive cultures among which 32 patients had Carbapenem-Resistant Enterobacteriaceae (CRE)/Carbapenem-Resistant Pseudomonas aeruginosa (CRPA) infection. The most common clinical syndrome in which the drug was used was occult sepsis (27/78; 34.6%) followed by primary bacteremia (20/78; 25.6%) and neutropenic sepsis (11/78; 14.1%). The clinical efficacy which was primarily assessed in terms of clinical cure was met for 55 (70.5%) patients. The 14-day mortality for the studies group was found to be 18 (23%).

Conclusion

The analysis of results shows encouraging clinical cure rates and 14-day mortality rates in a subset of severe infections which has limited treatment options.

## Introduction

Avibactam is a beta-lactamase inhibitor that has an action against class A, class C beta-lactamases, and some class D enzymes such as Oxa-48. The Infectious Disease Society of America (IDSA) and European Society of Clinical Microbiology and Infectious Diseases (ESCMID) declared the use of ceftazidime-avibactam (CEF-AVI) be restricted to confirmed Carbapenem-Resistant Enterobacteriaceae (CRE) or Carbapenem-Resistant Pseudomonas Aeruginosa (CRPA) infections and the ESCMID guidelines additionally included treatment of multidrug-resistant organism with limited treatment options (LTOs) [1,2]. In India, carbapenem resistance is highly prevalent in hospitals and there are limited antimicrobial options for such infections. CEF-AVI was approved in India for use in CRE infections while polymyxins are the preferred agents for empiric therapy in the critically ill. Polymyxins have limited use in the renally impaired and polymyxin B cannot be used in urinary tract infections [3]. CEF-AVI has shown superior efficacy over polymyxins in studies over varying sites of infection [4-6]. This study retrospectively evaluated the clinical outcomes of CEF-AVI use in the Indian population on various clinical syndromes with a high risk of Carbapenem-Resistant Organism (CRO) infection and LTO.

## Materials and methods

Study design

This was a single-center retrospective study conducted on in-patients who received CEF-AVI from January 2021 to December 2021. The protocol was approved by the ethics committee (IHEC no. 285). Patients were included in the study if (i) they received CEF-AVI alone or in combination with any other antibiotic including aztreonam for at least one day of therapy (DOT) and (ii) samples from relevant sites were sent for cultures before the start of antibiotic. Patients who were suspected or culture-confirmed to have urinary tract infections, intra-abdominal infections, or nosocomial pneumonia were excluded from the study. Also, patients were excluded if they could not be followed up for a minimum period of 14 days and those who did not receive one DOT of CEF-AVI.

Parameters and data collection

CEF-AVI was given as a 2.5g intravenous infusion over two hours thrice a day in a patient with normal creatinine clearance, dose adjustments for renally impaired patients were based on manufacturer recommendations, and the clinical condition of the patient, for example, a higher than recommended dosage was used in critically ill patients on hemodialysis/renal replacement therapy [7,8]. Indications were classified as empiric or targeted at the time of initiation of CEF-AVI. Empiric treatment with CEF-AVI was started if the patient had risk factors for CRE infections (CRE score) [9] or not responding to carbapenems without microbiological evidence but high suspicion of bacterial infection for the clinical deterioration. Clinical syndromes were classified into primary bacteremia, secondary bacteremia, occult sepsis, neutropenic sepsis, post-transplant sepsis, and skin-soft tissue infections (SSTI). Primary bacteremia, secondary bacteremia, and SSTI were microbiologically proven with blood or tissue cultures respectively whereas occult sepsis, neutropenic sepsis, and post-transplant sepsis were culture-negative diagnoses in their respective population as per relevant clinical guidelines [10-15]. In culture-negative clinical syndromes, such as occult sepsis, CEF-AVI was chosen based on the risk for CRE/CRPA infection and clinical judgment. Severity scoring was based on the third international consensus definition for sepsis and septic shock [8] and creatinine clearance was assessed based on the chronic kidney disease epidemiology collaboration (CKD-EPI) equation [16]. Positive cultures were identified and antimicrobial susceptibility was processed using the Vitek-2 system (bioMérieux, Marcy l’Etoile, France). Molecular resistance was detected either through Filmarray blood culture identification panel (bioMérieux, Marcy l’Etoile, France) for positive blood culture samples or Xpert Carba-R (Cepheid, France) testing from colonies of blood culture positive and non-blood culture samples with growth. The synergy between CEF-AVI and aztreonam was performed by Disc-E strip (Pfizer method as mentioned by the Indian Council of Medical Research) [17]. The time to initiation of CEF-AVI was the duration from the date of sample collection (at the time of suspicion of infection/sepsis) to the date of initiation of CEF-AVI.

Data were collected from electronic records and reviewed by study coordinators which included the patient’s demographic profiles, clinical and microbiological profiles, antimicrobial therapy characteristics, and patient outcomes. Combination therapy is the use of another antimicrobial along with CEF-AVI with or without aztreonam except for antimicrobials used for additional gram-positive coverage.

Outcomes and statistical analysis

The outcomes from this study were all-cause mortality at 14 days from the start of CEF-AVI and clinical response at seven days from initiation of the antibiotic. Clinical response was categorized as improved/not improved in terms of clinical/radiological/laboratory parameters or death within seven days of initiation of the drug. Secondary objectives were time to initiation of CEF-AVI, average DOT on CEF-AVI, and average dose per day of CEF-AVI.

Continuous variables - median with interquartile ranges or mean and categorical variables as frequencies. Comparison between all-cause mortality and clinical response with variables like clinical syndrome or combination therapy was done using chi-square tests/Fischer’s exact.

## Results

Eighty-three patients had received CEF-AVI within the study period and fit the study criteria. Five out of the 83 patients were lost to follow-up and discharged against medical advice and the remaining 78 patients were included in the study. The study population had a median age of 55.5 years and mostly comprised males (71.8%, 56 out of 78). Diabetes followed by systemic hypertension were the most common co-morbidities seen among the population and there was a likewise distribution of subjects based on the Charlson comorbidity index (CCI) as shown in Table 1. The average creatinine clearance was 81.49 ml/min/1.73 m^2^ with seven patients on hemodialysis during the duration of drug therapy.

**Table 1:** Demographics of patients * Severity scoring was based on the third international consensus definition for sepsis and septic shock. # Creatinine clearance was assessed based on the CKD-EPI (chronic kidney disease epidemiology collaboration) equation. $ Other antibiotics included minocycline, polymyxins, metronidazole, levofloxacin, and Fosfomycin.

Variables	Variables	Mean/median/number N=78	Percentage/range with interquartile range (IQR)
Age (years)		55.5	1-84; 28.8
Gender	Male	56	71.8%
	Female	22	28.2%
Comorbidities	Diabetes	35	44.9%
	Systemic hypertension	29	37.2%
	Coronary artery disease	18	23.1%
	Chronic liver disease	17	21.8%
	Chronic kidney disease	12	15.4%
Charlson comorbidity index (CCI)	No comorbidity (0)	5	6.4%
	Mild (1-2)	21	26.9%
	Moderate (3-4)	21	26.9%
	Severe (≥5)	31	39.8%
Clinical indications	Occult Sepsis	27	34.6%
	Primary Bacteremia	20	25.6%
	Neutropenic Sepsis	11	14.1%
	Post-Transplant Sepsis	7	9%
	Secondary Bacteremia	3	3.8%
	Skin and soft tissue infection	6	7.7%
	Miscellaneous	4	5.1%
Severity of infection*	No sepsis	27	34.6%
	Sepsis	31	39.7%
	Septic shock	20	25.6%
Creatinine clearance (ml/min/1.73 m^2^)^#^	<15	11	14.1%
	15-30	10	12.8%
	31-50	6	7.7%
	>50	51	65.4%
Combination therapy	Aztreonam	44	56.4%
	Other antibiotics^$^	18	23.1%

The most common clinical syndrome for which CEF-AVI was used was occult sepsis (34.6%) and followed by primary bacteremia (25.6%). Post-transplant patients included four post-bone marrow transplants and three post-liver transplant recipients. Miscellaneous syndromes include osteomyelitis, liver abscess, otitis media, and suspected meningitis constituting 5.13%, i.e., four out of 78 of the patients. The severity of infection ranged from no sepsis to septic shock (25.6%; 20 out of 78 patients).

**Figure 1:**
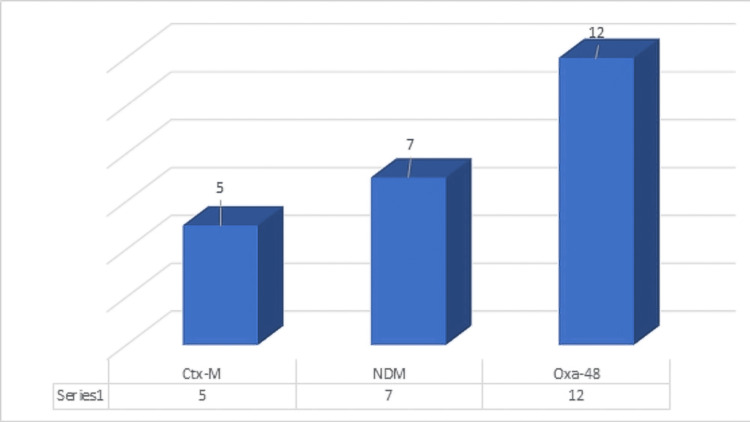
Molecular gene patterns among CRO isolates The above graph shows the incidence of molecular resistance genes among the isolates. N = 16; X-axis shows the resistance genes that were tested and Y-axis shows the incidence of those genes in the isolates. Three isolates harbored both oxa-48 and NDM genes and two among them were resistant to ceftazidime-avibactam and both showed synergy with aztreonam.

Antimicrobial therapy characteristics that were collected for the study included indication for initiation of CEF-AVI, microbiological characteristics, and combination with aztreonam or other drugs. Sixty-seven percent (52 out of 78) of the indications for initiating CEF-AVI were empiric and 33.3% (26 out of 78) were for targeted reasons. Forty-three (55.12%) patients out of 78 were culture positive. As few patients had multiple isolates totally there were 49 isolates in 43 patients. Thirty-five out of forty-nine isolates (71.4%) were CRO. Most are CRE (30 out of 49 isolates; 61.2%) and CRPA (five out of 49; 10.2%). One patient had an infection with both CRE and CRPA. Molecular resistance genes were analyzed in only 16 isolates and Oxa-48 was the predominant resistance gene detected (12 out of 16 isolates). CEF-AVI was resistant in nine (28.1%) out of 25 isolates in whom it was tested by the E-strip method. Table 2 shows the microbiological characteristics of this study and Figure 1 shows the molecular gene distribution among CRO isolates.

**Table 2:** Microbiological characteristics of patients * Distribution of sample types with growth - Urine and Bronchoalveolar lavage was sent for patients with occult sepsis and isolates were non-carbapenem-resistant organisms. # Other samples included bone, cerebrospinal fluid, liver abscess pus, and ear swab. ## The numbers in brackets() depict the number of isolates. $ Two patients had CRE isolated from two sites and thus the total number of isolates is 30. ** One patient had both Carbapenem-Resistant Enterobacteriaceae and Carbapenem-Resistant Pseudomonas Aeruginosa infection, so the total number of Carbapenem-Resistant Pseudomonas Aeruginosa isolates comes to 5. CRE: Carbapenem-Resistant Enterobacteriaceae; CRPA: Carbapenem-Resistant Pseudomonas Aeruginosa

Variables	Variables	Number of patients	Percentage of total (%)
Growth in culture (n=78)	Growth present	43	55.1
	No growth	35	44.9
Sample types* (n=43)	Blood	28	65.11
	Pus swab	2	4.6
	Tissue	4	9.3
	Urine	1	2.3
	BAL	4	9.3
	Others^#^	4	9.3
Pathogen isolated (n=43(49))^##^	CRE	28 (30)^$^	65.1 (71.4)
	CRPA	4 (5)**	9.3 (10.2)
	Others	11	25.6 (22.4)
Ceftazidime-avibactam susceptibility (n=25, 27)	Sensitive	16 (18)	59.3 (66.6)
	Resistant	9	3.6 (3.3)
Synergy with aztreonam (n=23(25))	Present	17 (19)	73.9 (68)
	Absent	6 (6)	26.1 (24)

CEF-AVI was resistant in nine out of 27 isolates that were tested and synergy testing between CEF-AVI and aztreonam was done in 25 out of 35 CRE/CRPA isolates which revealed that 76% of isolates had synergy between the two drugs. Aztreonam was given along with CEF-AVI in 44 patients (56.4%) out of the total 78 patients in the study. Other drugs given in combination included minocycline, polymyxins, metronidazole, levofloxacin, and Fosfomycin which formed 23.1% (18 out of 78) out of the total (Table 3).

Outcomes

**Figure 2:**
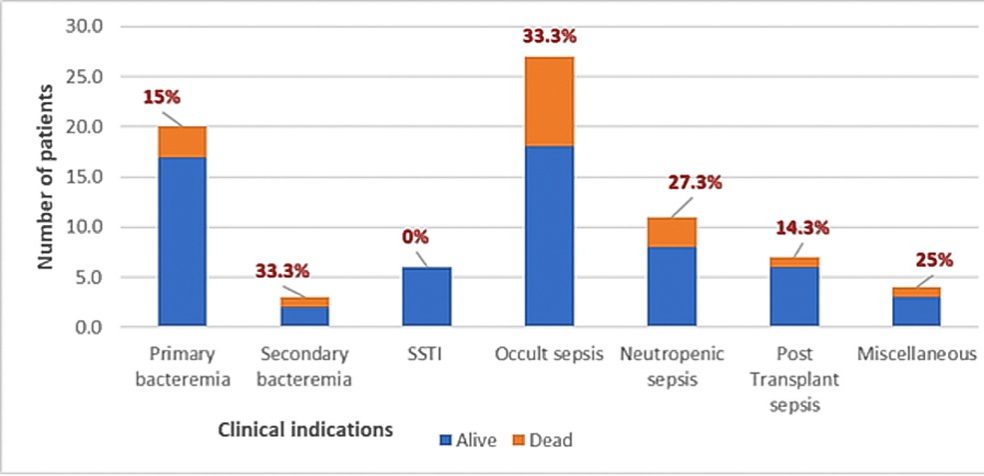
Proportion of mortality among the various clinical indication The above chart depicts the proportion of mortality (orange) among the various clinical indication (blue) in the total number of patients. Miscellaneous includes osteomyelitis, liver abscess, otitis media, and suspected meningitis. The percentage numbers in red depict the mortality rate in each clinical syndrome. SSTI: skin-soft tissue infections

Eighteen out of 78 patients (23%) in the study expired within 14 days of initiation of CEF-AVI and 55 patients (70.5%; 55 out of 78) improved clinically in seven days. Among the clinical indications treated with CEF-AVI, the highest proportion of death was observed among patients diagnosed with occult sepsis and secondary bacteremia (33%) then neutropenic sepsis (27.3%) (Figure 2). There was no significant difference between death among patients with culture growth or sterile cultures. There was no relation to mortality among patients receiving combination therapy with other antibiotics. Combination with aztreonam in 44 patients showed a 14-day mortality rate of 20.5% (nine dead out of 44). The median time to initiation of CEF-AVI was one day from sample collection and the median duration in terms of DOT for CEF-AVI is seven with a median average dose per day of 7.5 grams (Table 3).

**Table 3:** Outcomes of patients on ceftazidime-avibactam # Proportions/percentages have been taken with respect to the corresponding group and not the total sample size. * Combination of ceftazidime-avibactam with other antibiotics with or without aztreonam CEF-AVI: ceftazidime-avibactam

Outcome variable	Variable	Numbers/range	Proportion (%) or Median; IQR
Clinical Responses (in seven days)	Improvement	55	70.5%
	No improvement	23	29.5%
In Hospital Mortality (14 days)		18	23%
Mortality rate among variables^#^	Culture positive	10	23%
	Culture negative	8	22%
	Empiric	15	40.5%
	Targeted	3	11.5%
	Combination*	5	27.7%
	No combination	13	21.7%
	Aztreonam	9	20.9%
Time to Initiation (TOI) of CEF-AVI (days)		(0,18)	1; 3
TOI - Infection		(0,18)	3; 4
TOI - Sepsis		(1,5)	1; 3
TOI - Septic Shock		(0,4)	0.5; 2
Average dose per day of CEF-AVI (g/day)		(1.2,7.5)	7.5; 3.75
Average dose per day - Non-sepsis		(1.25,7.5)	7.5; 0
Average dose per day - Sepsis		(1.2,7.5)	7.5; 3.75
Average dose per day - Septic shock		(1.25,7.5)	7.5; 3.75
Average day of therapy with CEF-AVI (day)		(1,25)	7; 5
Average dose per day of AZTREONAM (g/day) N=44		(0.75-9)	6; 3

## Discussion

This was a retrospective study conducted on patients who received CEF-AVI in a quaternary care center in India. CEF-AVI is to be reserved as an option for CRE and CRPA infections, nevertheless, it is used empirically most often in critically ill patients with suspected hospital-acquired infections in our setting. This study investigated the use of the drug in non-Food and Drug Administration (FDA)-approved clinical syndromes, i.e., urinary tract infections, intra-abdominal infections, or nosocomial pneumonia as other studies have already been conducted among this group including one from our center [18]. Sixty-seven percent of patients enrolled in the study received the drug on an empiric basis and the most common clinical syndrome it was used for was in occult sepsis with no localizations or culture positivity. Seventy-seven percent of patients survived, with more than 70% showing clinical improvement on CEF-AVI. A retrospective study by Nagvekar *et al.* in India used CEF-AVI for the treatment of CRE infections and depicted a mortality rate of 21% similar to the present study [19]. Their study also had NDM and Oxa-48 gene prevalent in their isolates whereas our study had a predominance of Oxa-48 gene alone. Another study on CRE infections with a predominance of the Oxa-48 gene showed a much higher mortality rate of 50% in patients administered with CEF-AVI [20].

Most of the study population had sepsis/septic shock forming 65.3% of the total and CCI scoring was distributed evenly between mild (26.9%), moderate (26.9%), and severe (39.8%). Septic shock is associated with high mortality and the pharmacokinetics of most drugs cannot be relied upon in this situation. Other studies depicted good efficacy with varying percentages of patients with sepsis or septic shock and a study by Vena *et al.* demonstrated good clinical efficacy even when the majority of the study patients had septic shock [4,21,22].

The average dose per day as well as DOT of CEF-AVI in sepsis and septic shock remained comparable, also among 19 patients who had a creatinine clearance of less than 50 ml/min/1.73 m^2^. The time to initiation for CEF-AVI was lower in patients with septic shock (less than a day) and 50% (10 out of 20 patients) of these patients died within 14 days. The initiation of CEF-AVI within 48 hours of infection onset was found to be protective in a US study which was not observed here [23]. The median time to initiation in this study was one day and the least in the sepsis/septic shock group but calculated from the collection of samples and not the diagnoses of infection/sepsis.

Following the phase 3 trial of CEF-AVI in complicated intraabdominal infection by Mazuski *et al*., lower clinical cure rates were observed in the participants of the CEF-AVI arm with moderate creatinine clearance, this led to modifications in the dosing recommendations for renally impaired cases [24-26]. The present study used the earlier dosing recommendations by the manufacturer and patients who were critically ill were given higher dosing than recommended. Eighteen patients were given reduced dosing (excluding pediatric patients) and varied dosing with a median dose per day of 2.29g ranging from 1.25g every 48 hours to 1.25g thrice daily was used in hemodialysis. Thus, further analysis of this inconsistent variable was not made. The role that renally adjusted dosing played in mortality could not be assessed even though only four (22.2%) died out of the 22 patients who received the modified dosing regimen.

The study did not reveal any significant difference in mortality or clinical cure among patients with or without combination therapy with other antibiotics, which included minocycline, polymyxins, metronidazole, levofloxacin, and fosfomycin. This is in concordance with other studies where there was no reduction in mortality with combination therapy whereas, a study on critically ill patients with CRE infections showed a lower 30-day mortality rate [4,27,28].

Out of the 43 patients who were culture positive, 32 patients had CRE or CRPA infections. Thirty isolates from the study were CRE and five were CRPA. CEF-AVI testing was done in 27 isolates (25 patients), out of them nine showed resistance. Two of these nine isolates harbored both NDM and Oxa-48 genes, in whom synergy with aztreonam was present and all received a combination with aztreonam with no deaths. Synergy testing between CEF-AVI and aztreonam revealed six (24%) isolates having no synergy, with only the above two isolates resistant to CEF-AVI. Oxa-48 gene was the most commonly detected gene followed by NDM, this reflects the predominant resistance gene in the Indian population [29].

Limitations and further research

Being a retrospective study there were a lot of limitations with various variables not analyzed. The study did not evaluate adverse drug events or *Clostridium difficile-associated* diarrhea in the patients. Even though over 40% of isolates from these patients were CRE or CRPA, data on CEF-AVI sensitivity or synergy testing with aztreonam was limited. We also did not repeat cultures in culture-positive patients or assess for relapses/reinfections in them to watch for resistance development.

Further research needs to be carried out in our country for various clinical and microbiological indications for CEF-AVI. Studies on the evaluation of drug resistance development in strains where Oxa-48 and NDM resistance genes are prevalent need to be conducted. Even in this study, molecular analysis was not done on the CEF-AVI-resistant strains.

## Conclusions

The study revealed that in the use of CEF-AVI among patients with clinical indications that have limited antimicrobial treatment options, CEF-AVI has a good clinical cure rate and survival rate. Hence, CEF-AVI can be a promising option in treating various clinical syndromes which are at present not indicated by the FDA, where there is high suspicion of or confirmed multidrug organism infection with LTOs.
